# Guided Treatment Improves Outcome of Patients with Enterocutaneous Fistulas

**DOI:** 10.1007/s00268-012-1663-4

**Published:** 2012-06-06

**Authors:** Ruben G. J. Visschers, Wim G. van Gemert, Bjorn Winkens, Peter B. Soeters, Steven W. M. Olde Damink

**Affiliations:** 1 Department of Surgery, Maastricht University Medical Centre, NUTRIM School for Nutrition, Toxicology & Metabolism, Maastricht University, PO Box 5800, 6202 AZ Maastricht, The Netherlands; 2 Department of Methodology and Statistics and The School for Public Health and Primary Care, Maastricht University, P Debyeplein 1, 6229 HA Maastricht, The Netherlands; 3 Division of Surgery and Interventional Science, Royal Free Hospital, and University College London, Gower Street, London, WC 1E 6BT UK

## Abstract

**Background:**

The present study was designed to evaluate the effects of guided treatment of patients with an enterocutaneous fistula and to evaluate the effect of prolonged period of convalescence on outcome.

**Methods:**

All consecutive patients with an enterocutaneous fistula treated between 2006 and 2010 were included in this study. Patient information was gathered prospectively. Treatment of patients focused on sepsis control, optimization of nutritional status, wound care, establishing the anatomy of the fistula, timing of surgery, and surgical principles. Outcome included spontaneous and surgical closure, mortality, and postoperative recurrence. The relationship between period of convalescence and recurrence rate was determined by combining the present prospective cohort with a historical cohort from our group.

**Results:**

Between 2006 and 2010, 79 patients underwent focused treatment for enterocutaneous fistula. Cox regression analysis showed that period of convalescence related significantly with recurrence of the fistula (hazard ratio 0.99; 95 % confidence interval 0.98–0.999; *p* = 0.04). Spontaneous closure occurred in 23 (29 %) patients after a median period of convalescence of 39 (range 7–163) days. Forty-nine patients underwent operative repair after median period of 101 (range 7–374) days and achieved closure in 47 (96 %). Overall, eight patients (10 %) died.

**Conclusions:**

Prolonging period of convalescence for patients with enterocutaneous fistulas improves spontaneous closure and reduces recurrence rate.

## Introduction

The treatment of patients with enterocutaneous fistula (ECF) has proven to be extremely difficult throughout the past decades [1–6]. A previous report by the surgical department of the Maastricht University Medical Centre (MUMC) showed that guided treatment of a consecutive series of 135 patients resulted in favorable outcome [4]. Adherence to this guideline resulted in a closure rate of 87 %, mortality rate of 10 % [4], and a satisfactory quality of life [7]. A low preoperative albumin concentration (<25 g/L) was related to increased postoperative mortality, and sepsis was the main cause of death [4]. It was noticed that the restorative operation occasionally occurred before the predefined target period of convalescence of 6 weeks [4]. Reasons for nonadherence to the protocol were not always possible to obtain because of the retrospective nature of our study. In an editorial comment, it was suggested that prolonging the duration of convalescence may result in increased spontaneous closure rate [8].

The importance of the length of convalescence has been stipulated in earlier reports. Many specialized institutes adhere to a minimal period ranging from 6 weeks to 6 months [3, 4, 9]. Nevertheless, the most optimal interval between the occurrence of the fistula and reoperation has not been convincingly established [10, 11].

The MUMC has continued guided treatment of patients with an ECF. Based on our own data [4], we specifically adhered to a minimal period of convalescence of 6 weeks, aimed to operate on patients with an albumin concentration >25 g/L, and treated sepsis more aggressively. We hypothesized that this would lead to improved spontaneous closure and postoperative outcome, and reduce mortality in patients with an ECF. In this study, we evaluated the feasibility of the refined treatment guideline in a prospective cohort of consecutive patients with an ECF. We registered reasons for nonadherence to the protocol. We studied the effect of period of convalescence on recurrence rate and postoperative mortality by using our retrospective [4] and current prospective cohort of ECF patients. The effects of the guideline on recurrence rate, spontaneous and operative closure, and mortality was evaluated and compared with the previous patient series [4].

## Patients and methods

The MUMC is a tertiary referral center for patients with an ECF. All patients with an ECF consecutively treated at our institute between 2006 and 2010 were prospectively monitored. Patients with esophageal, gastroduodenal, pancreatic, biliary, and perianal fistulas were excluded. Patients underwent guided treatment focusing on the control of sepsis, optimization of nutritional status, wound care, establishing fistula anatomy, timing of surgery, and surgical strategy (SOWATS-protocol) as described in detail previously (Table 1) [4]. The most important adjustments from our previous retrospective evaluation included strict adherence to a minimal period of convalescence of 6 weeks, possibly allowing spontaneous closure in more patients, with the goal to perform surgery strictly when albumin levels are >25 g/L, and increased focus on controlling sepsis, because this was the main cause of death. Daily care and follow-up was guided by two surgeons (WG and SOD), a dietician, and a clinical nurse specialist. Patients were followed until March 2010 or death [4]. Patients were considered fit for restorative surgery when sepsis was controlled, adequate nutrition provided, edema resolved, and when serum albumin concentration was at least >25 g/L with normalizing leukocyte count and erythrocyte sedimentation rate (previously described in detail [4]). Sepsis was defined as fever, increased inflammatory parameters, decreased albumin concentration, positive fluid balance, edema, or organ failure in combination with suspected or documented infection [12].

**Table 1:** Treatment strategy in patients with an enterocutaneous fistula

Phase	Action
Sepsis control	Signs of sepsis
	Radiological drainage of abscess
	Relaparotomy on demand (minimally) invasive
	Consider other infectious foci; intravenous line, urinary tract, pulmonary
Optimization of nutritional status	Rehydration and electrolyte supplementation
	Enteral nutrition is preferred
	Parenteral nutrition to meet caloric requirements, small bowel ECF
	Allow 500 mL/day clear liquids orally
Wound care	Gauzes for low output ECF
	Collect ECF fluid with bag (wound manager/fistula bag), pastes to protect the skin
	Draining excessive ECF fluid with sump-suction
	Proton pump inhibitors
Anatomy of the ECF	Macroscopic
	Biochemical analysis of ECF fluid (bilirubin and amylase)
	Methylene blue
	Preoperatively; fistulography or contrast computed tomography: length of intestine and localization of origin of ECF, stenosis, obstruction and fluid collection
Timing of surgery	Clinically stable (above), psychologically willing to undergo surgery
	Albumin >25 g/L
	Period of convalescence >6 weeks
Surgical strategy	One-stage procedure
	Careful adhesiolysis
	Wedge excision or intestinal resection
	Limit number of anastomoses to minimum
	Cover sutures with healthy viable tissue
	Keep away from compromised area

Baseline variables were recorded, including age at the start of treatment (younger or older than 60 years), sex, primary disease (malignancy, inflammatory bowel disease or a miscellaneous group), cause (spontaneous or postsurgical), anatomy (small or large intestine), and output of the ECF (< or >500 mL/day), abdominal wall status (closed or defect), occurrence of a septic episode (yes or no), parenteral nutrition (PN: yes or no), and albumin concentration before restorative surgery (< or >25 g/L). Information about the restorative operation comprised duration of surgery (minutes), surgical technique (resection or no resection), and resorbable mesh insertion (yes or no). In case of closure of the abdominal wall with a double layer Vicryl mesh was used.

### Outcome parameters

The primary endpoint was recurrence of the ECF after the restorative operation, defined as renewed post-restorative connection between the intestine and the skin that did not close spontaneously. Secondary endpoints included spontaneous and surgical closure and mortality. Results of this prospective patient series were compared with our previously described historical cohort. Both patient series were combined to study the relationship between period of convalescence and recurrence and mortality. Postoperative complications were scored according to the classification by Dindo et al. [13], reporting the highest score for each patient.

### Statistical analysis

Differences were analyzed with the χ^2^ test for dichotomous parameters and analysed with the Mann–Whitney *U* test or Student’s *t* test, where appropriate, for continuous variables. The association between the duration of convalescence and recurrence of the ECF after elective surgery and postoperative mortality was determined with Cox proportional hazard analysis combining the prospective and historical patient cohort but still correct for database, i.e., differences between these prospective patient series and historical cohort. Proportional hazard assumption was checked with Schoenfeld residuals. Variables were checked for confounding and confirmed when more than a 10 % change of the hazard ratio occurred [14]. Two-tailed *p* values ≤0.05 were considered significant. All statistical analyses were performed using the Statistical Package for the Social Sciences (version 15; SPSS Inc., Chicago, IL).

## Results

### Patients

**Fig. 1:**
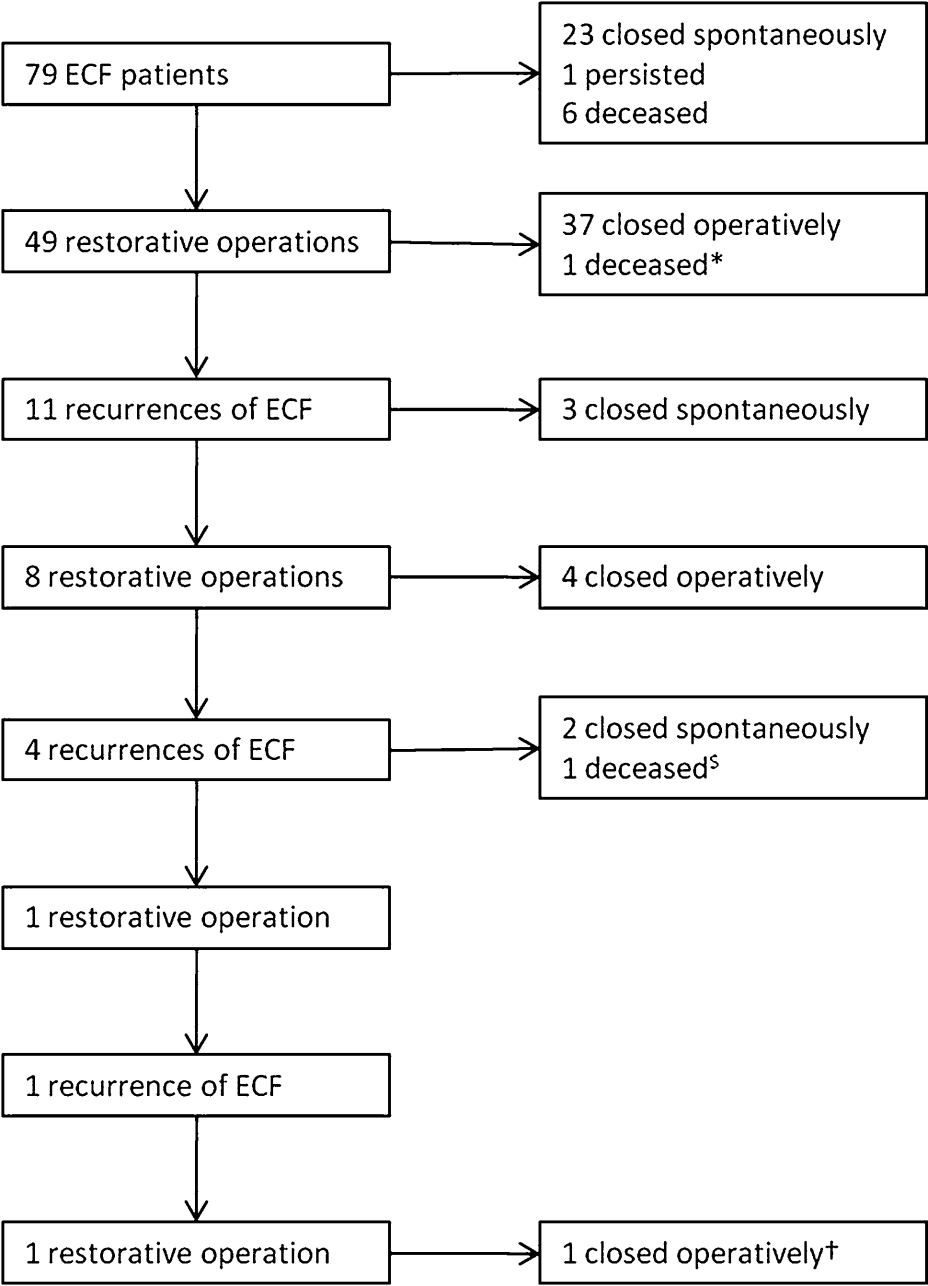
Flow chart of patients treated for their ECF. *Deceased 2 days after operative closure of the ECF, ^$^deceased 18 months after development of the third ECF, ^†^enterectomy and stoma of proximal jejunum

In total, 79 patients were consecutively treated for ECF at the MUMC between 2006 and 2010 (Fig. 1; Table 2). Patients were followed until March 2010 or death with a median of 24 (range 2–55) months. Forty-three (54 %) patients were referred from other hospitals. The median age of the patients was 59 (range 22–81) years, and 44 (56 %) patients were male. The population mainly consisted of patients with malignancy (*n* = 32, 41 %) or inflammatory bowel disease (*n* = 14, 18 %). The majority of patients developed the ECF after abdominal surgery (*n* = 68, 86 %). The present prospective series consisted of more patients with malignancy, more referrals, more low-output fistulas, and more patients receiving parenteral nutrition compared with the historical patient series (Table 2).

**Table 2:** Characteristics of patients ECF

Characteristics	2006–2009	1990–2005	*p* Value
	*n* (%)	*n* (%)	
Age (years)			
<60	37 (47)	67 (50)	0.78
≥60	42 (53)	68 (50)	
Sex			
Male	35 (44)	65 (48)	0.67
Female	44 (56)	70 (52)	
Primary disease			
Malignancy	32 (41)	30 (22)	0.01
IBD	14 (18)	24 (18)	
Other	33 (42)	81 (60)	
Source of referral			
MUMC	36 (46)	82 (61)	0.03
Other hospital	43 (54)	53 (39)	
Cause of fistula			
Spontaneous	11 (14)	16 (12)	0.62
Postoperative	68 (86)	119 (88)	
Anatomy of fistula^†^			
Small bowel	67 (85)	104 (80)	0.46
Large bowel	12 (15)	26 (20)	
Output of fistula (mL/day)^†^			
<500	54 (68)	56 (48)	0.005
≥500	25 (32)	61 (52)	
Abdominal wall^†^			
Closed	46 (58)	82 (61)	0.77
Defect	33 (42)	53 (39)	
Sepsis			
No	41 (52)	62 (46)	0.48
Yes	38 (48)	73 (54)	
Parenteral nutrition			
No	15 (19)	53 (39)	0.002
Yes	64 (81)	82 (61)	
Preoperative albumin (g/L)^a^			
<25	9 (18)	25 (29)	0.22
≥25	40 (82)	62 (71)	

^a^Determined in 49 patients undergoing a restorative operation in the prospective series and 97 in the historical series

^†^Numbers do not add up to total values as a result of missing values

### Period of convalescence

The historical and prospective patient series were combined to analyze the relationship between duration of convalescence and outcome. Between 1990 and 2010, 214 patients were treated with an ECF. In total, 148 patients were operated electively after a median of 72 (range 4–374) days. Twenty-four patients (16 %) developed a recurrent fistula after elective surgery (Table 3). These patients had been operated after a median duration of convalescence of 53 (range 7–302) days, which was significantly lower than patients without a recurrence (median, 81 (range 4–374) days; *p* = 0.033). Recurrences developed after a median of 14 (range 2–319) days after closure of the first ECF (Fig. 1). Cox regression analysis showed that period of convalescence related significantly with recurrence of the ECF (hazard ratio (HR) 0.99; 95 % confidence interval (CI) 0.98–0.999; *p* = 0.04; Table 3). There were no confounding variables. Eleven patients (7.4 %) died postoperatively. Duration of convalescence was not related to postoperative mortality (HR 1.007; 95 % CI 0.996–1.014; *p* = 0.242). Costs were not higher with increased period of convalescence but did relate to recurrence. Patients with a recurrent fistula had a prolonged need for parenteral nutrition, increased number of diagnostic procedures, and operative interventions, which resulted in increased costs of total treatment €71,904 (standard error (SE) €9,274) versus €48,964 (SE €5,776) for patients with nonrecurrent ECF (*p* = 0.008).

**Table 3:** Cox regression analysis of recurrence rate of the enterocutaneous fistula

Characteristic	Total	Patients with recurrence (%)	HR (95 % CI)	*p* Value
Age (years)				
<60	72	12 (17)	1	0.75
≥60	76	12 (16)	0.879 (0.394–1.963)	
Sex				
Female	81	16 (20)	1	0.15
Male	67	8 (12)	0.532 (0.227–1.247)	
Primary disease				
Malignancy	42	7 (17)	1	0.91
IBD	27	5 (19)	0.916 (0.4–2.775)	0.92
Other	79	12 (15)	0.683 (0.397–4.094)	0.68
Cause of fistula				
Spontaneous	20	1 (5)	1	0.16
Postoperative	128	23 (18)	4.184 (0.565–30.989)	
Source of referral				
Other hospital	72	12 (17)	1	0.98
MUMC	76	12 (18)	0.989 (0.443–2.207)	
Anatomy of fistula^a^				
Small bowel	120	20 (17)	1	0.81
Large bowel	26	4 (15)	1.144 (0.384–3.409)	
Output of fistula (mL/day)^a^				
<500	72	8 (11)	1	0.21
≥500	60	11 (18)	1.807 (0.724–4.509)	
Abdominal wall^a^				
Closed	80	11 (14)	1	0.42
Defect	68	13 (19)	1.395 (0.624–3.118)	
Sepsis				
No	78	12 (15)	1	0.49
Yes	70	12 (17)	1.335 (0.592–3.008)	
Parenteral nutrition				
No	61	8 (13)	1	0.76
Yes	87	16 (18)	1.146 (0.483–2.716)	
Preoperative albumin (g/L)^a^				
<25	30	6 (20)	1	0.11
≥25	99	13 (13)	0.467 (0.183–1.187)	
Period of convalescence (days)				
–	–		0.99 (0.98–0.999)	0.04

Univariate Cox regression analysis

*IBD* inflammatory bowel disease

^a^Numbers do not add up to total values as a result of missing values

### Conservative treatment

Conservative treatment of the ECF was first designed to stabilize the patient by controlling sepsis, providing adequate nutritional support, and proper wound care.

#### Sepsis control

Sepsis was diagnosed in 38 (48 %) patients, usually heralding the development of ECF. CT imaging proved the presence of an intra-abdominal abscess as the primary focus in 26 of these patients. Radiologically guided drainage was possible in 11 of these patients, whereas 15 patients required operative drainage. A central venous line infection was considered the primary source of sepsis in six patients and consequently needed replacement. Six patients had already developed abdominal sepsis in the referring hospital and received treatment there. Sepsis was controlled in almost all patients (92 %) except three who died due to uncontrollable sepsis despite CT-guided (*n* = 2) and operative drainage (*n* = 1). Additionally, 14 patients had no signs of sepsis but did have an intra-abdominal abscess of whom 13 underwent radiological drainage and 1 patient required surgical drainage. Eight patients were admitted to the intensive care for a median of 3 (range 1–60) days.

#### Optimization of nutritional status

PN was supplemented from the onset of the ECF in 64 (81 %) patients for a median of 39 (range 7–230) days and a total of 2,888 PN days. Patients received omega-6 long chain fatty acids (Intralipid^®^ 20 %, Fresenius Kabi N. V., Utrecht, NL) as standard lipid emulsion. In all cases, a tunneled double-lumen Hickman was preferably inserted into the internal jugular or subclavian vein.

#### Wound care

A specialized nurse delivered wound care, particularly in patients with a high-output ECF and/or abdominal wall defect. A wound manager or fistula bag was used to collect fistula fluid in 33 patients. A drain with low suction residing in a wound manager achieved adequate wound care in 28 patients. A drain inserted into the fistula tract immediately removed the fistula fluid in 11 patients. Gauzes sufficed to collect fistula fluid in seven patients with low output ECFs and a closed abdominal wall.

#### Anatomy of the ECF

Various techniques were used to diagnose the ECF and to specify the anatomical origin. In some patients, the intestine already could be partially observed in the abdominal wound or macroscopic analysis of fistula fluid was possible. Bilirubin and amylase concentrations in wound fluid were measured in 17 patients, radiological examination was performed in 24, and a combination of both was used in 38 patients. In ten of the aforementioned, the ECF was diagnosed after oral ingestion of methylene blue. In 14 patients, radiological examination was performed by fistulography and CT imaging in the other 48. Radiological imaging of the ECF and abdomen occurred in almost all patients undergoing a restorative operation (*n* = 45, 92 %), all ultimately by computed tomography.

#### Outcome of conservative treatment

Because of the high risk of postoperative complications, as shown after previous intra-abdominal operations, we refrained from performing a restorative operation in one patient, allowing the ECF to persist. An indwelling drain made the ECF properly manageable for this patient. Ultimately, conservative management achieved spontaneous closure in 23 (29 %) patients (compared with 19 patients (16 %) in the previous series, *p* = 0.023) after a median period of convalescence of 39 (range 7–163) days (Fig. 1) compared with median of 18 (range 7–49) days; *p* = 0.007). Conservative closure was achieved predominantly in patients with a closed abdominal wall and/or large bowel ECF.

### Operative treatment

#### Timing of surgery

Spontaneous closure was considered unlikely in 49 (62 %) patients (with abdominal wall defect and/or small bowel fistula). These patients underwent a restorative procedure after a median period of 101 (range 7–374) days (compared with a median of 53 (range 4–270) days in the historical group, *p* < 0.001; Fig. 1). One patient had to be operated on before the predefined target period of 6 weeks because of fecal peritonitis and uncontrollable sepsis (compared with 33 patients of 107 from the historical series, *p* < 0.001). Nine patients were operated on with an albumin concentration <25 g/L. In six of these patients, albumin concentration was stable for several weeks without any signs of sepsis. However, these patients were not thought to improve any further but were considered to be at risk of developing sepsis or becoming malnourished even further. Three patients with low preoperative albumin required emergency surgery because of fecal peritonitis (*n* = 2) and volvulus (*n* = 1). All patients with a low preoperative albumin concentration survived with successful closure of the ECF.

#### Surgical procedure

All patients underwent a one-stage restorative operation predominantly performed by the same team of surgeons (WG and SOD). The median duration of surgery was 260 (range 60–690) minutes. A complete adhesiolysis was performed in all patients. A fistulectomy with intestinal minimal segment resection (<5 cm) was performed in 15 patients, and an additional intestinal resection was performed in 34. Twenty-two patients received an absorbable mesh to close the abdominal wall. It was always possible to cover the anastomosis with healthy matrix (i.e., omentum and/or loops of bowel). An intra-abdominal drain was always left in situ temporarily (less than 48 h), and skin was always closed.

#### Postoperative closure

Eleven patients showed new signs of a fistula after a median of 8 (range 2–319) days after closure of the ECF, which resolved spontaneously in three. All eight (16 %) patients in whom the ECF recurred (similar percentage to historic control) were operated on again. Thereafter, four patients developed another ECF. One patient with severe Crohn’s disease developed three recurrences after which an enterectomy could not be avoided despite optimal medical treatment. Ultimately, the ECF remained closed in 47 (96 %) patients who underwent surgical restoration, leading to overall closure in 70 (89 %) patients.

#### Postoperative morbidity

Thirty-seven (76 %) patients developed a postoperative complication. Thirteen patients (26 %) only required medical or radiologically guided treatment, whereas 24 patients (49 %) required surgical intervention and/or needed intensive care. Two patients had a functional short bowel and remained dependant on PN after treatment.

#### Mortality

Six (8 %) patients died during conservative treatment as a result of progressive sarcoma, Merkel cell tumor, cardiogenic shock (occluded coronary arteries), pancreatitis, and a complicated course after sepsis with pulmonary embolism and concurrent vascular insufficiency resulting in weaning problems and cachexia. The sixth patient suffered from cystic fibrosis and was initially treated for sepsis in our center but died after referral to a transplantation center due to uncontrollable sepsis. One patient required an emergency operation on day 120 because of an occlusion of the superior and inferior mesenteric artery and died 2 days postoperatively. One patient died in another hospital as a result of a pulmonary embolism 18 months after development of the second recurrence. Thus, two patients (4 %) died postoperatively, amounting to an overall mortality of the initial cohort of 10 %. In-hospital mortality was 6 (8 %) and 1-year mortality was 7 (9 %).

## Discussion

Adherence to a treatment guideline for patients with an ECF can lead to favorable outcome. Yet, considering these data, it appears that it is sometimes difficult to adhere to the protocol, e.g., when acute medical treatment is required, which stresses the importance of individual patient assessment. Patience before embarking on surgery allows increased spontaneous closure. Recurrence rate and mortality did not change significantly over time. However, postponing surgical treatment and allowing improvement of patient’s health status improves postoperative outcome as evidenced by a reduction in recurrence rate.

Low preoperative albumin concentrations have been associated with increased mortality [4, 6, 15]. Therefore, our goal was to operate on patients when albumin concentration was <25 g/L. However, three patients required an emergency operation because of acute sepsis as a result of fecal peritonitis. More importantly, six patients underwent a restorative operation with low albumin concentration without acute indication. Yet, these patients suffered from malignancies or severe Crohn’s disease, which impeded full recovery of their health status, including increase of albumin level [16, 17]. Therefore, these patients were considered at risk of developing infectious complications and becoming malnourished, despite optimal treatment. Consequently, the window of opportunity to operate on these patients was considered small, and ultimately, after a period of convalescence of 6 weeks, patients underwent a successful restorative operation.

The prospective series includes more patients with malignancy, more small-bowel fistulas, more patients receiving PN, and fewer patients with a high output fistula compared with our previous report. It must be considered that these changes have contributed to the differences in outcome as well. Unfortunately, this cohort is too small to perform a multivariable analysis.

Evaluation of patients with an ECF treated at the MUMC between 1990 and 2005 showed that we not always adhered to the minimal period of convalescence of 6 weeks, leaving less chance for spontaneous closure [4, 8]. It has become clear that spontaneous closure most likely occurs in a closed abdominal wall, warranting prolongation of the period of convalescence in these patients [4, 18, 19]. This prolongation has allowed the period during which spontaneous closure could occur to double, successfully increasing the spontaneous closure rate from 16 to 29 %. One should primarily aim for spontaneous closure of the ECF, thus avoiding surgery. This occurs most likely in patients with a closed abdomen, low output ECF, and an uncomplicated disease course [3, 4, 20].

Postoperative mortality was 4 % and compared well with most recent results reported by other specialist units [21, 22]. In contrast to what we expected, overall mortality rate remained 10 %, indicating a shift from postoperative to preoperative deaths. This suggests that preoperative selection criteria have been adequate but that there is a group of patients who ultimately succumb to the combined stress of their primary disease and ECF, and in whom a restorative operation will offer no solution, neither early nor late. This would imply that we are reaching the limits of our possibilities to cure certain patients with an ECF. Although the number of patients studied is small, it can be suggested that a more aggressive approach to abdominal sepsis as shown in this prospective series results in adequate control. Subsequently 3 of 8 patients died from sepsis, whereas it had been 10 of 13 in the historical series. Future studies should put more emphasis on the variables related with development of the ECF, providing possible options for prevention.

Because of the difficulty and high risk of operative treatment of the ECF, nonoperative options have been explored to achieve definitive closure. Although some have advocated the use of a vacuum-assisted closure device for open abdominal treatment of ECF [23], it has been suggested recently by Fisher that this technique can be counterproductive, because it might induce new fistulas that seem to have a higher mortality [24]. The use of low-vacuum suction devices, as used for patients in the present study, offers a good tool to collect fistula fluid, although it did not induce spontaneous closure, nor did we see renewed fistulization. Fibrin glue has been introduced for the treatment of perianal fistulas. Although it is minimally invasive and easy to use, it is associated with a high recurrence rate in patients with perianal fistulas [25]. Avalos-Gonzalez et al. [26] studied a fibrin sealant in patients with low-output gastrointestinal fistulas, showing increased closure time without increase in spontaneous closure, although additional studies remain imperative. Somatostin and its analogues have shown no significant increase in spontaneous closure [27, 28]. To conclude, the use of a TNF-alpha inhibitor, infliximab, also has been shown to be advantageous for closure ECFs in patients with Crohn’s disease [29]. The effect of this drug in non-Crohn’s ECF remains to be determined.

In conclusion, standardized treatment of ECF patients achieves good results. Prolongation of the period of convalescence is associated with increased spontaneous closure and a reduced recurrence rate after surgery. Although overall mortality has remained stable, postoperative mortality seems to be reduced, implying an improved preoperative screening. Future studies should focus on the prevention of fistula development.
